# Beyond Maternal Tolerance: Education of Uterine Natural Killer Cells by Maternal MHC Drives Fetal Growth

**DOI:** 10.3389/fimmu.2022.808227

**Published:** 2022-05-10

**Authors:** Delphine M. Depierreux, Jens Kieckbusch, Norman Shreeve, Delia A. Hawkes, Bryan Marsh, Robert Blelloch, Andrew Sharkey, Francesco Colucci

**Affiliations:** ^1^ Department of Obstetrics and Gynaecology, University of Cambridge School of Clinical Medicine, NIHR Cambridge Biomedical Research Centre, Addenbrooke’s Hospital, Cambridge, United Kingdom; ^2^ Centre for Trophoblast Research, University of Cambridge, Cambridge, United Kingdom; ^3^ Department of Urology, University of California, San Francisco, San Francisco, CA, United States; ^4^ Department of Pathology, University of Cambridge, Cambridge, United Kingdom

**Keywords:** reproductive immunology, pregnancy, uterine natural killer, education, fetal growth, *β*2m

## Abstract

Reproductive immunology has moved on from the classical Medawar question of 60 years ago “*why doesn’t the mother reject the fetus?*”. Looking beyond fetal-maternal tolerance, modern reproductive immunology focuses on how the maternal immune system supports fetal growth. Maternal uterine natural killer (uNK) cells, in partnership with fetal trophoblast cells, regulate physiological vascular changes in the uterus of pregnant women and mice. These vascular changes are necessary to build the placenta and sustain fetal growth. NK cell functions in the uterus and elsewhere, including anti-viral and anti-tumour immunity mediated mostly by blood NK cells, are modulated by NK cell education, a quantifiable process that determines cellular activation thresholds. This process relies largely on interactions between self-MHC class I molecules and inhibitory NK cell receptors. By getting to know self, the maternal immune system sets up uNK cells to participate to tissue homeostasis in the womb. Placentation can be viewed as a form of natural transplantation unique in vertebrates and this raises the question of how uNK cell education or missing-self recognition affect their function and, ultimately fetal growth. Here, using combinations of MHC-sufficient and -deficient mice, we show that uNK cell education is linked to maternal and not fetal MHC, so that MHC-deficient dams produce more growth-restricted fetuses, even when the fetuses themselves express self-MHC. We also show that, while peripheral NK cells reject bone marrow cells according to the established rules of missing-self recognition, uNK cells educated by maternal MHC do not reject fetuses that miss self-MHC and these fetuses grow to their full potential. While these results are not directly applicable to clinical research, they show that NK education by maternal MHC-I is required for optimal fetal growth.

## Introduction

Natural killer (NK) cells clear diseased cells, secrete immune mediators and regulate placentation. NK cell receptors (NKR) can be inhibitory or activating and provide a balance of signalling cues upon engagement with their ligands on neighboring cells and thus ensure appropriate responses (1). Some of these NKR, such as human KIR and mouse Ly49 are expressed stochastically resulting in a variegated repertoire of NK cell subsets with diverse functional potentials and activation thresholds (2–5). NK cells in different tissues are also heterogeneous in terms of the expression patterns of other receptors, including integrins and adhesion molecules. Conventional NK cells (cNK) are characterized by expression of the integrin CD49b (i.e. DX5) and are the major NK cell in the blood and spleen. Tissue resident NK cells typically express CD49a and are found in many organs including the liver, skin, lungs, gut and in the pregnant and non-pregnant uterus (6). Strikingly, NK cells in the decidua (dNK) can account for up to 70% of leukocytes during early human pregnancy, and up to 30% in early mouse pregnancy (7, 8). Uterine NK cells in both human and mouse comprise discrete subsets, which can broadly be divided into dNK1, dNK2 and dNK3 in humans and type 1 innate lymphoid cells (ILC1), conventional NK (cNK), and tissue-resident (trNK) cells in mice (9–12).

The hypothesis that NK cells recognise abnormal cells by their lack of self-molecules, known as the missing self-hypothesis (13), was elegantly demonstrated in 1990 using the first ever KO mice, which carried an homozygous deletion for β2 microglobulin (*β*2m), the accessory molecule necessary for cell surface expression of all MHC class I molecules by human and mouse cells (14, 15). *β*2m KO bone marrow cell-transfer failed to reconstitute WT mice immuno-depleted by irradiation, while WT bone marrow cell-transfer did. Recipient mice rejected the *β*2m KO bone marrow cells because these failed to express MHC class I molecules and therefore could not engage inhibitory NKR on host NK cells. This experiment was key to proving the missing-self hypothesis correct (13). In early 2000’s, the concept of NK cell education (also called licensing) was formulated after discovering that peripheral NK cells lacking self-specific inhibitory receptors were hyporesponsive (16–18). This proposed that NK cells expressing self-specific inhibitory receptors become functional because they can also be inhibited and therefore their response is tightly controlled. NK cells that do not express such receptors fail to reach their functional potential and become anergic to avoid potential auto-immune reaction. Hitherto, the mechanism(s) through which NK cells become educated remains unclear. One model hypothesizes that NK cells are initially unresponsive and become functional during development *via* interaction of their NKR with self MHC class I (16). A second model suggests, in contrast, that NK cells are initially responsive, but become anergic as they are chronically stimulated by self-MHC class I expressed by healthy cells (19). In both cases, NK cell functional potential is finely tuned by the number of self- MHC class I inhibitory NKR and their affinity for self-MHC I (20). Current evidence suggests that NK cell education is not static and can be rapidly tuned by changes in local MHC levels (21, 22).

Although NK cell education was described in 2005, its physiological relevance has remained elusive. Education is not essential for murine NK cell responses to viral or bacterial infection and uneducated NK cells can sometimes function more effectively than their educated counterparts (23–25). However, we demonstrated recently that NKG2A-mediated education of uNK improves pregnancy outcome in mice and humans (26). Others have recently shown a role for NK-cell education in responses to viral infections in mice (27, 28).

**Figure 1:**
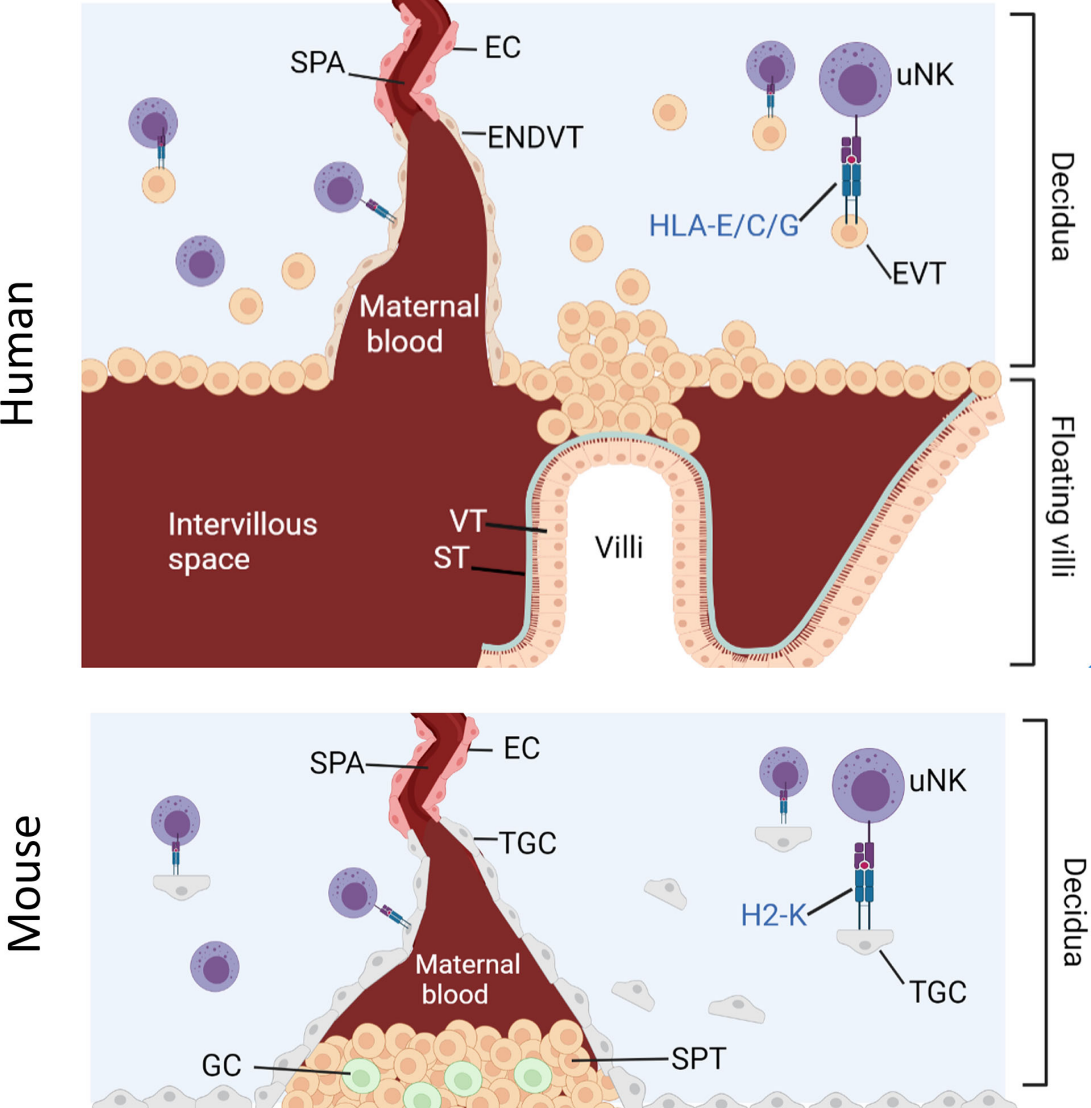
Interaction between uNK cells and HLA class I positive cells at the feto-maternal interface. Humans: Spiral arteries develop through the decidua and fill the intervillous space with blood that supplies the placenta. Placenta villi are lined with villous cytotrophoblast (VT) under a layer of syncitiotrophoblast (ST), both of which are HLA class I and HLA class II negative. The villi are attached to the decidua *via* fetal extravillous trophoblast (EV). Some EVT migrate and line the spiral arteries as endovascular trophoblast (ENDVT) and other EVT invade the decidua where they interact with maternal lymphocytes such as dNK cells. Both EVT and ENDT express non classical HLA class I -E/C/G but do not express classical HLA- class I -A/-B and are HLA class II negative. Mice: Invasive trophoblast giant cells (TGC) from fetal origin form the boundary of the implantation site. They invade the maternal decidua and remodel the spiral arteries (Spa) by displacing the endothelial cells (EC) and allow the maternal blood flow through the spongiotrophoblast (SPT) layer to the labyrinth (not shown here). The spongiotrophoblast (SPT) and the glycogen cells (GC) are the major endocrine compartment of the placenta. In B6 mice, TGC lack expression of non-classical MHC-I but express classical MHC-I H2-K which can interact with maternal lymphocytes in the decidua such as uNK cells.

NK cell education during pregnancy is unique because uNK cells are exposed to both maternal and fetal MHC-I molecules (29). Human and mouse placentation is characterized by the invasion of fetal trophoblast cells into the maternal decidua where they encounter maternal uNK (**Figure 1**). Extravillous trophoblast (EVT) in humans and trophoblast giant cells (TGC) in mice express an unusual array of MHC molecules. EVT expresses HLA-C, HLA-E and HLA-G whilst murine TGC express H2-K (30–33). This MHC-I repertoire seems to engage with receptors on cells of the innate immune system, including NK cells and macrophages. Interactions between NKR and maternal or paternal MHC class I molecules can therefore affect uNK cell functions through both education and inhibition.

Despite the difference between mice and human placentation, uNK are major lymphocytes in the decidua in both species and both EVT and TGC can interact directly with uNK. Given the limited availability of human samples, the mouse model is a useful tool to study the role of uNK cells in pregnancy. Because it is possible to engineer mice that completely lack MHC-I expression, the contribution of paternal from maternal MHC-I to uNK education on reproductive success can be dissected and the hypothesis that excessive inhibition of uNK by paternal MHC-I impairs reproductive success can be tested.

Evidence from human and mouse studies supports the idea that uNK cells regulate placental development and hence fetal growth (26, 34–37). Although mice lacking fully functional NK cells are fertile and produce litters of normal size, fetal weights are lower, possibly as a consequence of insufficient transformation of uterine arteries seen in these models (38–40). Fetal growth is tightly controlled and even small alterations from normal growth trajectories come at a cost; low birth weight is associated with increased susceptibility to cardiovascular disease and metabolic syndrome through fetal programming (41). This risk is further increased by deviations from the normal postnatal growth trajectory, due to ‘catch-up’ neonatal growth (42, 43).

**Figure 2:**
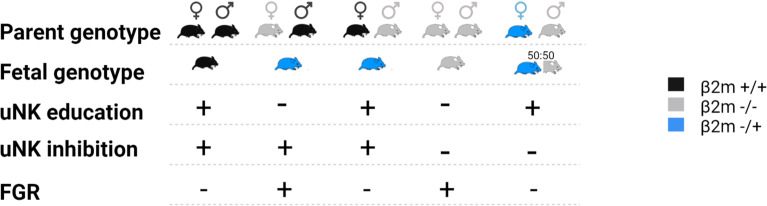
Representation of the crosses used in this study and impact on the fetal growth restriction (FGR). Females and male mice expressing either two alleles of *β*2m (black), one (blue) or none (grey) were mated in various crosses. The resulting fetal genotype is indicated according to the same color code. Whether the dam uNK cells were educated *via* maternal self-MHCI or inhibited via fetal MHC-I is indicated with plus or minus symbol. The impact of each cross on fetal growth restriction is indicated on the bottom line where a + indicates presence of FGR and – its absence.

Here, we used mice lacking MHC-I expression to examine the contribution of paternal and maternal MHC-I to uNK education. This model allows the effects of education on uNK functions and reproductive success to be dissected separately from the effects of excessive inhibition of uNK when they encounter paternal MHC-I expressed on fetal TGC. We examined uNK function and fetal growth using the same mouse model that was used to demonstrate the missing-self hypothesis; so *β*2m^-/-^ mice, and *β*2m+^/-^ heterozygous mice are compared to wild-type (WT) C57BL/6 (B6) controls. While cells of WT and heterozygous *β*2m^+/-^ mice have MHC-I on their surface, those of homozygous *β*2m^-/-^ mice lack it. To isolate the effect of maternal and fetal MHC on either education or uNK inhibition by MHC class I molecules, we generated crosses where either one or both parents and/or the conceptuses lack MHC class I molecules (**Figure 2**). We show that education dependent on maternal MHC class I molecules is essential for uNK cell functions because it programs uNK cells to drive fetal growth. The hyporesponsiveness of uneducated uNK cells resulted in reduced uterine arterial remodeling and increased incidence of fetal growth restriction (FGR), followed by rapid postpartum catch-up growth. FGR was also observed in pregnancies in which uNK cells were both uneducated by maternal MHC-I and not inhibited by trophoblast MHC-I, suggesting that lack of inhibition when uNK encounter trophoblast does not compensate for lack of education in uNK cells. This contrasts with peripheral NK cell responses to infection (25). Our results provide further evidence of the physiological importance of NK cell education and they confirm the link between uNK cell education with both female reproductive fitness and health of the offspring.

## Results

### Enhanced Functional Responsiveness of Educated uNK Cells

**Figure 3:**
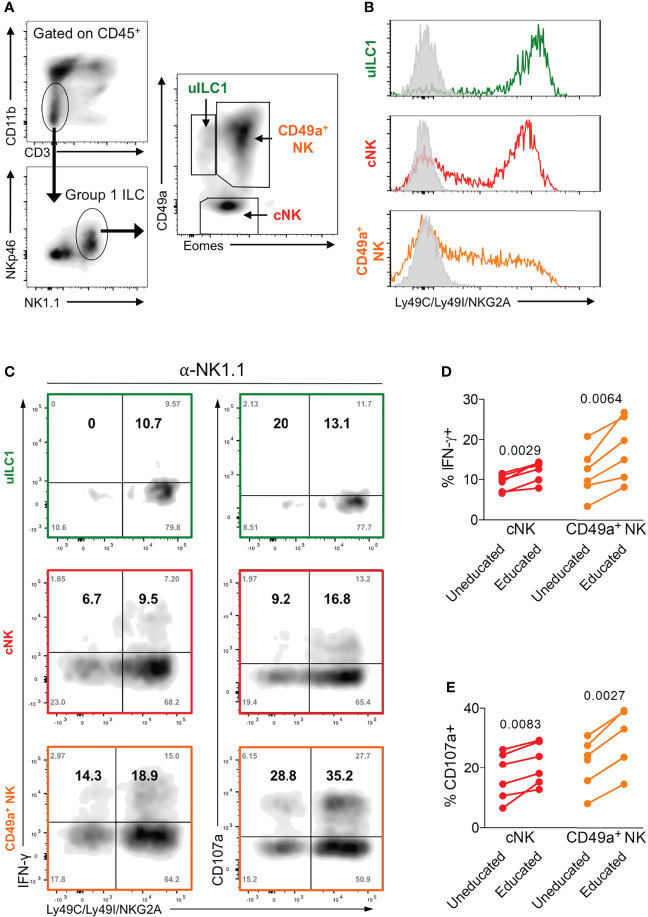
Enhanced function of educated mouse uNK cells. **(A)** Gating strategy for uterine group 1 ILC and delineation into three subsets including uterine ILC1 (uILC1), conventional NK cells (cNK) and tissue-resident CD49a^+^ NK. **(B)** Representative staining of all three group 1 ILC subsets for self-receptors in C57BL/6 mice. cNK, trNK or uILC1 subsets were stained for Ly49C/I, and NKG2A. Coloured line shows proportion of each subset positive for one or more of these educating inhibitory receptors. Grey line indicates staining with isotype matched negative control antibody. **(C)** Assessment of the functional responsiveness (intracellular IFN-γ and surface CD107a) after crosslinking by plate bound anti-NK1.1 antibody in cells expressing inhibitory NK receptors for self MHC compared to those that do not. Shown are the % of cells in each quadrant (grey text in corners) as well as the relative percentage of responders among cells expressing receptors for self and responders that do not have self-receptors (in bold). The relative percentage in the top left (Q1) and right (Q2) quadrants were calculated from the raw values as follows: Q1/(Q1+Q3) and Q2/(Q2+Q4). **(D, E)** Enumeration of IFN-γ producing **(D)** and CD107a^+^ NK cells **(E)** among cells expressing at least one receptor for self MHC class I (educated subset, self-receptor +) and those that do not (uneducated, self-receptor -). uILC1 are not depicted due to the paucity of uneducated cells in this subset. Data representative of 3 **(A, B)** or 2 **(C-E)** experiments with n = 6 mice per group. *P*-values in **(D, E)**, comparing cells in cNK or CD49a^+^ NK subsets expressing an educating self-receptor with those that do not, within each mouse using paired two-tailed Student’s t-tests.

NK cells are typically identified as CD3^-^ NKp46^+^ NK1.1^+^ cells in B6 mice (44, 45). In the uterus, both trNK cells (Eomes^+^ CD49a^+^), which are uniquely found in the uterus, and cNK cells (Eomes^+^ CD49a^-^), which are similar to mouse peripheral NK cells, express T-bet and produce IFN-γ, which is the key NK-produced cytokine in mice that drives arterial remodelling necessary for optimal placentation and fetal growth (12, 46, 47) (**Figure 3A**). In addition to uterine trNK and cNK cells, tissue-resident uILC1 (T-bet^+^ Eomes^-^ CD49a^+^) also produce IFN-γ. We have previously shown that mouse uterine cNK cells are educated by maternal MHC class I molecules (48). Here we wanted to determine the effect of education on the functions of tissue resident uNK cells and uILC1. To do this, we analysed the expression of educating inhibitory NKR for self MHC class I molecules on all three cell types. The major educating NKR in B6 mice (H-2^b^ MHC background) are Ly49C (preferentially binds H-2K^b^), Ly49I (binds H-2D^b^), and NKG2A (binds Qa-1^b^) (49). Large fractions of tissue-resident uNK cells, uterine cNK cells and uILC1 express at least one of these self-specific NKR at mid-gestation (**Figure 3B**). To investigate the functional consequences of NK education, we compared NK cell responsiveness upon *ex vivo* crosslinking of the activating receptor NK1.1, in cells expected to be educated or not based on their NKR expression profile (**Figure 3C**). The expression of NK1.1 is equivalent in educated and uneducated NK cells (**Figure S1**). In both trNK and cNK cell subsets, cells expressing inhibitory receptors for self-MHC class I responded with a greater percentage of IFN-γ^+^ or CD107^+^ cells, demonstrating their ability to be educated by their MHC environment (**Figures 3D, E**). There were too few uneducated uILC1 for this comparison. These results show that the interaction of maternal MHC class I molecules with inhibitory NKR educates both trNK and cNK cells in the uterus and unlocks their full functional responsiveness.

### Effect of Maternal MHC Class I Molecules on uNK Cell Development and Function

**Figure 4:**
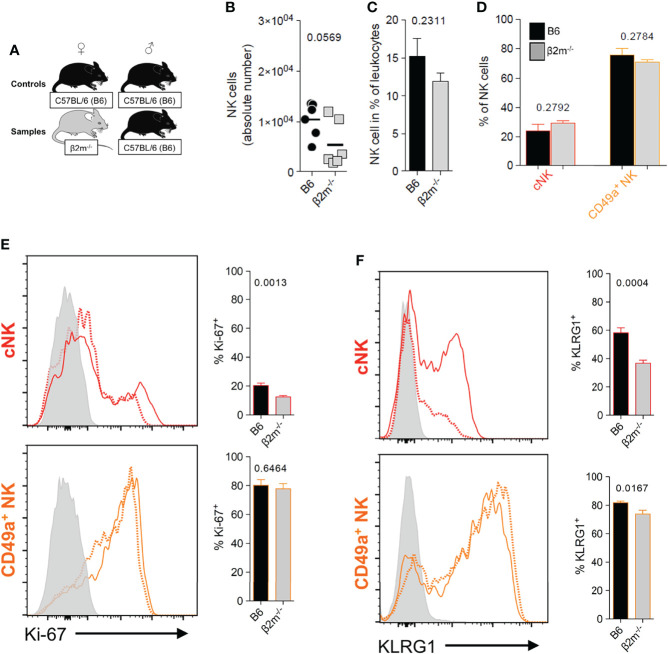
Maternal MHC class I molecules are dispensable for the development of uNK cells. **(A)** Mating strategy to assess the impact of MHC class I on uNK by comparing wildtype females with *β2m^-/-^* mice that lack surface expression of MHC-I, both mated with B6 males. **(B, C)** Comparison of abundance of all uNK cells in terms of absolute number per implantation site **(B)**, and relative frequency among CD45^+^ cells **(C)**. uNK numbers were measured at gestational age Total uNK were gated as shown in **Figure 3A**. **(D)** Relative abundance of the 2 main subsets of uNK within NK1.1^+^ NKp46^+^ Eomes^+^ cells. **(E, F)** Phenotypic assessment of uNK for Ki-67 **(E)** which is associated with cell proliferation and KLRG1 which marks terminal maturation and correlates with education in cNK cells **(F)**. Data representative of 3 experiments with n = 6-7 mice per group. Means ± SEM. *P*-values from unpaired two-tailed Student’s t-tests. See also **Figure S1**.

To better understand the downstream effects of uNK cell interactions with maternal MHC class I molecules during placentation, we used *β2m^-/-^* mice, which have a C57BL/6 (B6) genetic background. These mice lack cell surface expression of classical (H-2K and H-2D) as well as non-classical (Qa-1) MHC class I molecules (**Figure S2B**), and their trophoblast cells differ from those of B6 mice which do express H2-K and, to a lesser extent H-2D (30–33, 50) (**Figures S2C–D**).Their NK cells are thus uneducated but can be activated by cytokines (16) and respond well to cytomegalovirus infection (51). The frequencies and numbers of uNK cells in *β2m^-/-^* mice were not significantly different to those in wild-type B6 mice (**Figures 4A–D**). We stained for KLRG1 and Ki-67 to assess terminal maturation and proliferation of cells *ex vivo*. The cNK cell pool in *β2m^-/-^* mice contained lower frequencies of mature (KLRG1^+^) and proliferating (Ki-67^+^) cells compared to controls. The pool of CD49a^+^ uNK cells had also reduced frequencies of mature KLRG1^+^ but comparable frequencies of proliferating Ki-67^+^. cells (**Figures 4E, F**). Thus, maternal MHC class I molecules are not required for the development of uNK cells, but their absence affects maturation and proliferation of cNK cells in the uterus, which may be a consequence of failed education. In contrast, lack of MHC class I molecules did not affect phenotype or development of uILC1 (**Figure S3**).

**Figure 5:**
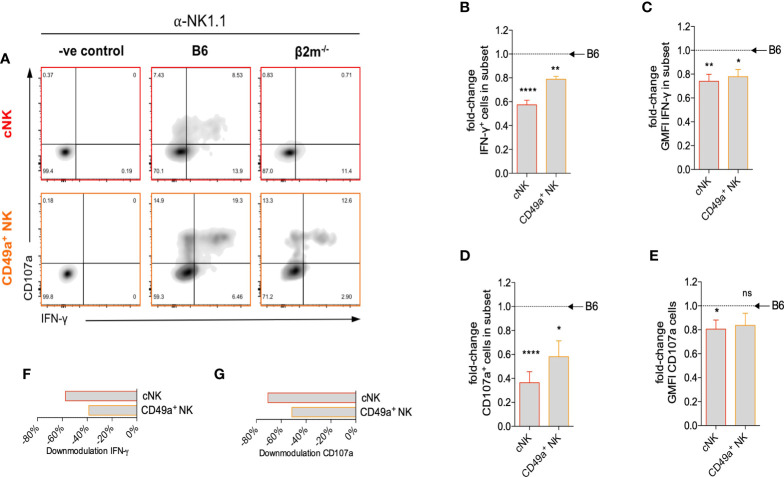
Surface expression of maternal MHC class I molecules is necessary for uNK cell function. **(A)** Representative density plots for detection of intracellular IFN-γ and surface CD107a on uNK subsets from *β2m^-/-^* or B6 controls at gd10.5 after crosslinking *in vitro* with α-NK1.1. Gates were set on samples where the antibodies for both parameters were omitted. **(B)** Fold-change in fraction of each subset of uNK from *β2m^-/-^* mice that is positive for IFN-γ compared to B6 controls. **(C)** Fold-change in mean fluorescence intensity among IFN-γ^+^ uNK compared to B6 controls. **(D)** Fold-change in fraction of subsets of uNK positive for CD107a compared to B6 controls. **(E)** Fold-change in mean fluorescence intensity among CD107a^+^ uNK compared to B6 controls. **(F, G)** Downmodulation of IFN-γ **(F)** and CD107 **(G)** in uNK cells. This metric combines the number of cells responding and the reduction in GMFI, as indicated in the methods. Data representative of three independent experiments with n=5-8 mice per group. Means ± SEM. *P*-values from unpaired two-tailed Student’s t-tests. GMFI, geometric mean fluorescence intensity. See also **Figure S2**. *p<0.05 , **p<0.01, ****p<0.0001. ns, not significant.

NK cell-derived IFN-γ plays an important role in the transformation of the arteries during pregnancy in mice (40). We next studied how the lack of NK education in *β2m^-/-^* mice affected IFN-γ production by uNK cells. After NK1.1 crosslinking, both the percentage of cells producing IFN-γ (**Figures 5A, B**) and the mean amount of IFN-γ produced per cell (**Figure 5C**) were reduced in both subsets of uNK cells in *β2m^-/-^* mice. IFN-γ production by uILC1 remained largely unaffected, **Figure S4**. The percentage of CD107a^+^, a proxy for degranulating cells, was also reduced in both uNK subsets in *β2m^-/-^* mice (**Figures 5D, E**). Thus, similarly to peripheral NK cells, both cNK and CD49a+ uNK subsets require interaction with MHC class I molecules to achieve normal responsiveness (**Figures 5F, G**). In the absence of MHC-I expression, cNK responses are more significantly downregulated than CD49a^+^ uNK cells (**Figures 5F, G**).

### Absence of Maternal MHC Class I Molecules Affects Uterine Arterial Morphology

**Figure 6:**
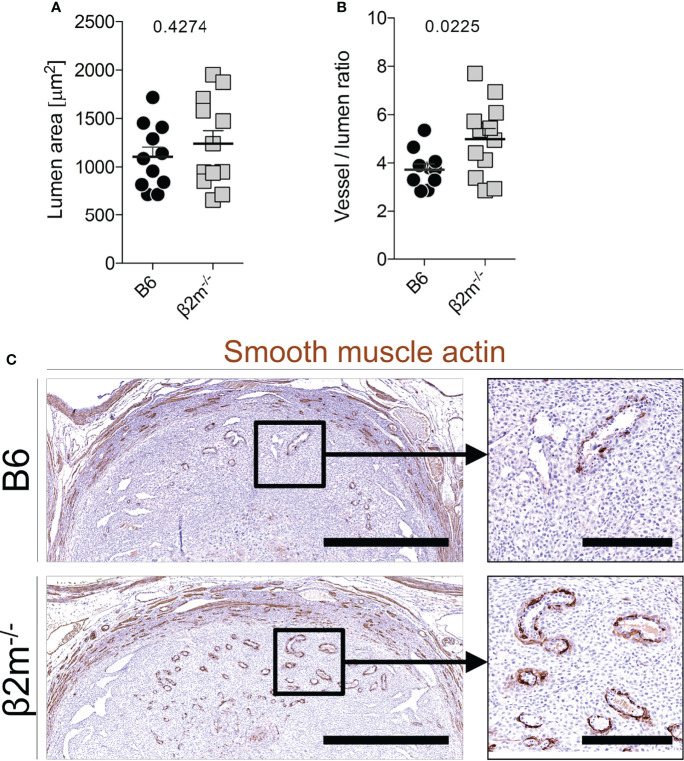
Reduced arterial remodelling in females lacking MHC class I** (A, B)** Quantitative assessment of blood vessel size **(A)** and relative vessel wall thickness **(B)** at gd9.5 in mice of the indicated genotype (all mated with B6 males). Data representative of n=11-12 conceptuses from 4 litters each. **(C)** Qualitative assessment of disappearance of smooth muscle actin at gd9.5 by immunohistochemistry. Bars = 1mm (left panel), 250µm (right panel). Means ± SEM. *P*-values from unpaired two-tailed Student’s t-tests. See also **Figure S3**.

During the normal transformation of uterine arteries, medial smooth muscle is lost with dilatation of the vessel, and this process is dependent on the presence of NK cells (39, 52). We assessed how arterial transformation is altered in the absence of educated NK cells. Although the size of the spiral artery lumen at midgestation in *β2m^-/-^* mice did not change (**Figure 6A**), the relative thickness of the smooth muscle media was greater, suggesting higher arterial stiffness (**Figure 6B**). In addition, actin within the media was retained in *β2m^-/-^* mice (**Figure 6C**). These findings correlated with less detectable IFN-γ in the decidua and myometrium of *β2m^-/-^* mice in 3/5 pregnancies (**Figure S5**). Taken together, these results show that the absence of educated uNK cells is associated with failure to undergo normal arterial remodelling during murine pregnancy.

### Fetal Growth Restriction in Pregnancies Where Maternal uNK Cells Are Uneducated

**Figure 7:**
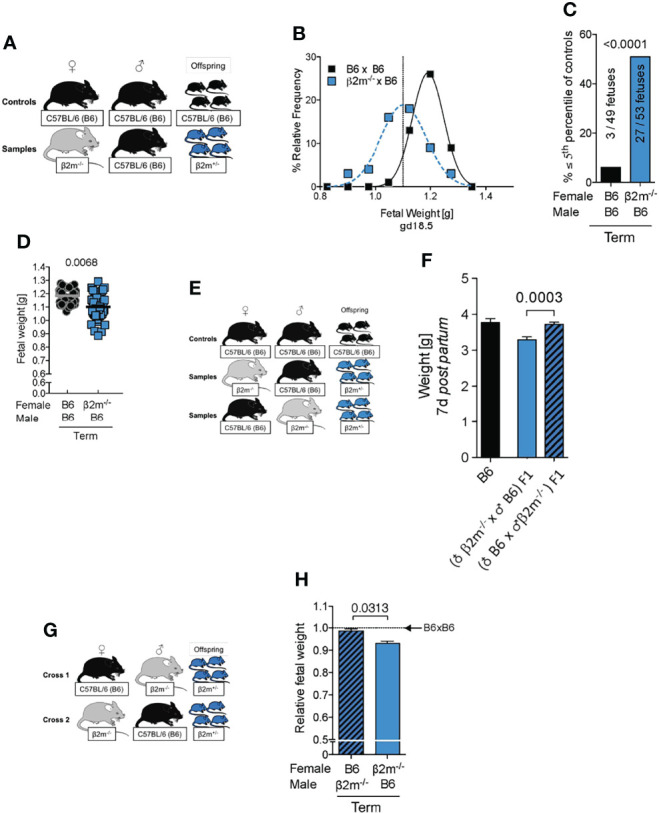
Fetal growth restriction in pregnancies where mothers lack MHC class I molecules. **(A)** Mating strategy to assess the impact of maternal MHC class I expression on fetal growth. **(B)** Weight distribution of fetuses carried by females with or without MHC class I surface expression. Dashed vertical line demarcates 5^th^ percentile of B6 controls. **(C)** Categorical analysis of fetuses within or below the 5^th^ percentile of controls. *P*-value from Fisher’s exact test. **(D)** Comparison of mean fetal weights. *P*-values from a mixed model analysis taking into account the clustering of observations by gestational age and/or litter. *P*-values above data points are indicative of comparison between crosses on the given time point. **(B-D)** data representative of 42 – 59 fetuses from 6 – 8 litters per time point per cross. **(E)** Mating strategy to assess the effect of maternal MHC class I surface expression on *post partum* growth of isogenic offspring compared to homozygous wildtype controls. **(F)** Comparison of weight on *post partum* day 7 between isogenic F1 fetuses with maternal or paternal *β2m^-/-^* compared to wildtype B6 controls. Means ± SEM, data representative of 17 – 54 pups per group from 4 – 7 litters. *P*-value from an unpaired two-tailed Student’s t-test. **(G)** Mating strategy to control for fetal genotype. **(H)** Comparison of isogenic fetuses carried by females with or without MHC class I surface expression relative to B6 controls. n = 46-68 fetuses from 7 – 9 litters per group. Fetal weight measured at term. *P*-value from a mixed model analysis taking the clustering of observations by litter into account.

Low birth weight followed by rapid catch up growth *post-partum* predisposes to a range of morbidities including premature death (42), reduced cognitive function (53), cardiovascular conditions and metabolic syndrome (41). To determine how lack of education of maternal uNK cells affected birth weight in our mouse model, we assessed the distribution of fetal weights at term and determined the incidence of fetal growth restriction using the 5^th^ percentile of controls as a cutoff (**Figures 7A-C**). Weights of fetuses carried by *β2m^-/-^* mothers with uneducated uNK cells were shifted to the lower end of the spectrum with a >8-fold increase in very small fetuses (<5^th^ percentile). This was also reflected in a moderate, but robust change in mean fetal weight (**Figure 7D**). Reduced growth was not limited to the intrauterine period but extended to at least day 7 *post-partum* (**Figures 7E, F**). However, the weights of newborn mice born from dams with uneducated uNK cells caught up during the second week of life (not shown). The reduction in birth weights was not associated with changes in the number of viable offspring (data not shown). We conclude that education of uNK cells is required for normal fetal growth. Because the fetuses in our model lack only one allele of *β2m*, they should in principle have a normal growth potential. To test this, we used the reverse cross, i.e. wild-type B6 females mated with *β2m^-/-^* males (**Figure 7G**). Heterozygous fetuses in this cross showed normal growth (**Figure 7H**), demonstrating that FGR in *β2m^-/-^* mothers was due to the maternal, not the fetal genotype.

### Fetal Growth Restriction in *β2m^-/-^* Mice Cannot Be Reversed by Removing Inhibition of Maternal uNK Cells

We have shown before that increased uNK cell inhibition by paternal MHC class I on trophoblast impedes fetal growth both in mice (48) and humans (54). It is thus possible that uNK cell inhibition contributes to reduced fetal growth in *β2m^-/-^* dams carrying *β2m^+/-^* fetuses, because the latter do express MHC and can therefore inhibit maternal uNK cells. To establish the contribution of uNK cell inhibition by MHC on trophoblast, we used a mating combination where MHC is missing in both mother and fetus (*β2m^-/-^* females x *β2m^-/-^* males). In this cross, maternal uNK cells will be uneducated but also disinhibited by the lack of MHC on trophoblast. In this setting, uneducated uNK cells could be activated by the missing self MHC on trophoblast, and thus drive fetal growth, despite the lack of education. However, we found that FGR persisted in this setting (**Figure S6**), emphasising the importance of uNK education for full functional responsiveness. This is in contrast to uneducated peripheral NK cells which do respond to either cytokine stimulation *in vitro* (16), or infection *in vivo* with both cytomegalovirus and *Listeria monocytogenes* (25, 51, 55, 56). These results also excluded the possibility that the few residual CD8^+^ T cells in *β2m^-/-^* mice could contribute to FGR by responding to paternal MHC on trophoblast (57).

### Educated Maternal uNK Cells Do Not Target Feto-Placental Units That Lack Self MHC

**Figure 8:**
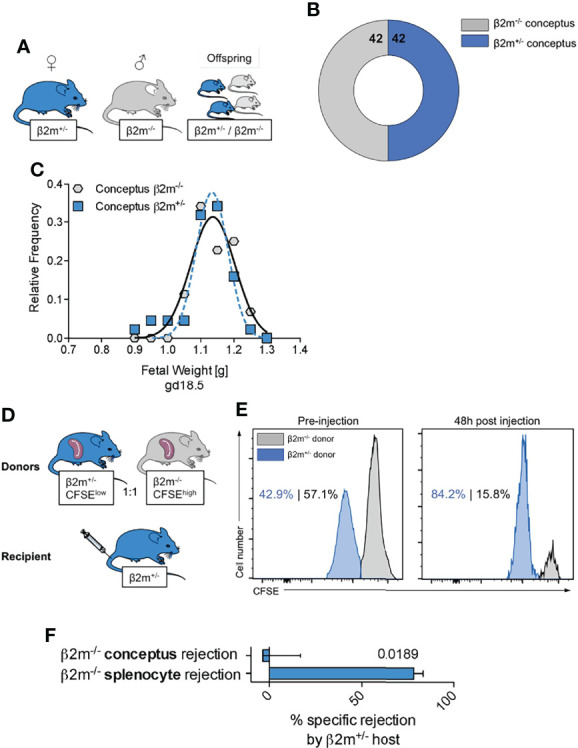
Educated uNK cells do not harm fetuses lacking self MHC class I** (A)** Mating strategy to assess the effect of absent placental/fetal MHC class I expression in MHC sufficient hosts. **(B)** Enumeration of fetuses either sufficient or deficient for MHC class I surface expression carried by MHC sufficient females. **(C)** Weight distribution of fetuses either sufficient or deficient for MHC class I surface expression carried by MHC sufficient females. Data representative of 11 litters. **(D)** Experimental layout to assess ability of heterozygous *β*2m^+/-^ mice to reject MHC class I-deficient haematopoietic cells (splenocytes). **(E)** Representative histograms showing *in vivo* rejection of MHC class I deficient splenocytes by MHC class I sufficient hosts. **(F)** Comparison of specific rejection of MHC-deficient conceptuses and splenocytes. Means ± SEM. Data representative of 84 offspring **(A-C)** and 5 mice per experiment **(D-F)**. P-value from an unpaired two-tailed Student’s t-test. CFSE, Carboxyfluorescein succinimidyl ester.

One consequence of uNK cell education is the generation of NK cell subsets that can efficiently sense and remove cells not displaying the normal array of self MHC class I molecules (58). Our mouse model offers the opportunity to assess the consequences of the interactions between educated uNK cells and fetal trophoblast cells lacking all MHC class I molecules. When mated with *β2m^-/-^* males, heterozygous *β2m^+/-^* females will have educated uNK cells that interact with both *β2m^+/-^ and β2m^-/-^* fetal trophoblast cells in the same litter. Loss of the inhibitory signal to uNK cells resulting from the absence of paternal MHC class I molecules in *β2m^-/-^* placental cells might trigger activation of uNK cells by missing-self recognition, which could result in either fetal loss or altered growth in *β2m^-/-^* embryos, compared to their *β2m^+/-^* litter-mates (**Figure 8**). *β2m^-/-^* cells do express equivalent amounts of ligands for activating receptors NKG2D and DNAM-1 (**Figure S7**) and so are able to activate NK cells. We found, however, the same number of *β2m^+/-^ and β2m^-/-^* fetuses and these had similar weights in 11 litters tested (**Figure 8B, C**). These results show that educated uNK cells are not ‘hyperactive’ towards *β2m^-/-^* embryos and their placentas, but instead drive normal fetal growth. In contrast, peripheral NK cells in *β*2m^+/-^ mice do reject transplanted splenocytes from *β*2m^-/-^ mice (**Figure 8D-F**) (59).

## Discussion

A long-standing conundrum in NK cell biology is that the same inhibitory signals can regulate both education and inhibition of NK activation. Despite extensive investigation, the molecular basis and physiological relevance of NK education remain uncertain. The remodelling of the lysosomal compartment in NK cells is a plausible mechanism to reconcile education and inhibition. The inhibitory signal would let granule contents accumulate during education, upon inhibitory NKR ligation by self MHC, and then, when MHC is missing, the granule content would be powerfully released to kill the missing-self target (60, 61). While this is an elegant explanation, it may not pertain to uNK cells, which have low cytotoxic activity. Here, we set out to resolve the relative contribution of the two processes of education and inhibition on uNK cells during pregnancy.

Our study shows that education by maternal MHC class I molecules enhances uNK cell responsiveness in mice and promotes fetal growth. Absence of an educating maternal NKR/MHC interaction leads to hypofunctional uNK cells *in vitro* and it affects arterial transformation *in vivo* (**Figure S8**). Furthermore, neither uneducated nor educated uNK cells seem to be activated by missing-self recognition of *β2m^-/-^* fetal trophoblast cells encountered in the decidua. To a certain extent, our findings differ from previous data in immunity to infections and cancer obtained using peripheral NK cells, where MHC class I education might be redundant (51). Uneducated NK cells can be even more effective than educated NK cells in fighting viral infections (25). This suggests that NK cell education may have tissue-specific functions.

Whilst the impact on fetal growth may seem subtle, it is likely to have biological relevance as fetal growth restriction is closely tied to lifetime health outcome. In our mouse model lacking all MHC-mediated educating interactions of NK cells, more than half of fetuses did not reach their genetic growth potential in any given litter. This is not only disadvantageous for the fitness of offspring at the time of birth, but is also detrimental because the rapid, compensatory catch-up growth that follows predisposes to morbidity (53, 62) and early death (42, 43). Thus, maternally educated uNK cells contribute to an optimal start in life. Further, we detected no obvious morphological abnormalities of placentas carried by *β*2m^-/-^ (data not shown), suggesting rapid compensation. We previously showed that lack of education *via* CD94/NKG2A also results in FGR, but placental weight was unaffected in NKG2A deficient dams and the placental transcriptome showed limited alterations (26). FGR in *β*2m^-/-^ females is probably due to faulty uterine arterial remodeling, a process known to be mediated by uNK cell-derived IFN-γ (40). Retention of smooth muscle actin in the vessel walls and increased wall thickness found previously have been shown to affect resistance index and blood flow characteristics in uterine arteries, which will affect the essential transfer of nutrients and oxygen (26).

Our study has also allowed us to investigate the long-standing issue of how much paternal MHC-I molecules contribute to uNK education. Both human and murine studies show that excessive inhibition of uNK by paternal MHC-I expressed on invading fetal trophoblast impairs reproductive success (34). Mice expressing a single additional MHC class I molecule on fetal trophoblast, that engages more inhibitory NKR on maternal uNK than in wild-type mice display FGR and reduced uterine arterial remodelling (48). However, it has been difficult to ascertain whether the effects of paternal MHC-I on trophoblast result from direct inhibition or effects on education of maternal uNK cells. Comparison of fetal growth in B6 and *β*2m^-/-^ dams both bearing *β*2m^+/-^ offspring clearly shows that uNK education by maternal MHC affects fetal growth. Indeed the interactions of inhibitory maternal NKR with paternal MHC are the same in B6 and *β*2m^-/-^ dams, however only the latter, who fail to educate their uNK cells, experience fetal growth restriction.

The implications of these findings for human pregnancy is not yet clear. To understand the contribution of uNK education to pregnancy outcome, we will need to pinpoint the molecular mechanisms regulating uNK cell function. Human genetic studies show that certain genetic combinations of maternal NKR and fetal HLA class I molecules that favour strong uNK inhibition, associate more frequently with pregnancy complications such as pre-eclampsia, recurrent abortion and low birth weight (29, 48, 54, 56, 63). It therefore seems paradoxical that almost all uNK cells express at least one inhibitory receptor for self MHC class I, something not seen in other tissues. But if one considers that uNK cell education might confer an advantage to uNK cells, then the ubiquitous expression of inhibitory NKR by uNK cells may explain this paradox. On the other hand, it may be that the presence of at least one inhibitory NKR (i.e. NKG2A) on 95% uNK cells, may secure maternal tolerance of the fetus. Future work could include studying the relative contribution of the different uNK and ILC1 populations to the phenotype we observed.

Several important questions remain unanswered. It is not clear how uNK cell education impacts on human reproduction. In women, uNK cells are educated by interactions between their inhibitory receptors and MHC, but the effects of specific receptor/ligand combinations suggest NK education may differ between tissue and blood NK cells (64). We have recently shown that genetically determined NKG2A-education in humans confers a small but significant advantage in the process leading to development of pre-eclampsia, because women genetically programmed to use the HLA-E-NKG2A pathway are exposed to a 7% smaller relative risk for this condition (26). This provides indirect evidence that uNK education may also impact the outcome of pregnancy in humans.

More broadly, another area that has not been extensively studied is the contribution of NK education by MHC-I independent pathways, whether education of uNK cells occurs *via* interaction in -cis or in -trans, or a combination of both, and finally, the intracellular pathways and mechanisms leading to NK education also remain elusive, although SHP-1, a cytosolic protein involved in NK cell inhibition signalling, appears to be an important mediator of the process (65–67).

Overall, our results clearly demonstrate that maternal education is key to normal uNK function. They contribute to further our understanding of NK cell education in the context of pregnancy. As well as the previously described effects of inhibitory interactions of NKR binding to MHC-I on trophoblast (68), these results show that education of uNK by maternal MHC-I is also required for a successful pregnancy.

## Methods

### Mice

All animal experiments were approved by the University of Cambridge Ethical Review Panel and carried out in accordance with Home Office Project License PPL 70-8222. C57BL/6 (B6) mice were purchased from Charles River UK. Mice with *B2M ^tm1Unc^* targeted mutation (*β2m^-/-^*) have been described before (14) and were backcrossed to B6 background for >10 generations. Mice were 8–12 weeks of age. For pregnancy experiments, female mice were randomly introduced to males and the timing of conception was determined by detection of a copulation plug representing gd0.5. Number of mice used are as in the following: **Figure 3**: data representative of 3 (A+B) or 2 (C-E) experiments with n=6 mice per group; **Figure 4**: data representative of 3 experiments with n=6-7 mice per group; **Figure 5**: data representative of three independent experiments with n=5-8 mice per group; **Figures 6A, B**: Data representative of n=11-12 conceptuses from 4 litters each; **Figures 7B-D**: data representative of 42 – 59 fetuses from 6 – 8 litters per time point per cross; **Figure 7F**: data representative of 17 – 54 pups per group from 4 – 7 litters; **Figure 7H**: n=46-68 fetuses from 7 – 9 litters per group; **Figure 8**: data representative of 84 offspring (A-C) and 5 mice per experiment (D-F).

### Cell Preparation and Functional Assays

Cells suspensions of mouse spleens and uterine tissues were prepared as described previously using Liberase TM (69) or DH (47) (both Roche). Generally, the Liberase TM protocol was used for enumeration and phenotyping of cells as it does not introduce a bias through a density gradient. The Liberase DH protocol was used for functional assays to discard dead cells prior to incubation. For *in vitro* stimulation, cells were cultured on plates pre-coated with 10 μg ml^−1^ anti-NK1.1 for 9.5 h (PK136, BioLegend). Brefeldin A and monensin (eBioscience, 1x concentration) were added 1 h after the start of the experiment. Samples were acquired on an LSRFortessa (BD) and analysed using FlowJo (Treestar). Downmodulation of effector functions was calculated according to the following formula: -100% x [1-(fold-change_responders_ x fold-change_GMFI_)].

### Flow Cytometry

Cells were typed using biotin- or fluorochrome-conjugated antibodies with specificity for CD45 (30-F11), NK1.1 (PK138), CD11b (M1/70), CD49a (HMα1), CD49b (DX5), IFN-γ (XMG1.2), KLRG1 (MAFA), CD3ϵ (145-2C11), NKp46 (29A1.4), CD107a (1D4B), and Ki-67 (B56), Eomes (Dan11mag), Ly49C (4LO3311), Ly49I (YLI-90), NKG2A (16a11), PVR (TX56), B2M (S19.8), H2-Db (KH95), H2-Kb (AF6-88.5), Qa1-b (6A8.6F10.1A6), pan-cytokeratin (C11), Rae pan (REA273), purchased from BD PharMingen, BioLegend, Invitrogen, Miltenyi or eBioscience. Clone 4LO3311 was a gift by Jennifer Laurent. Dead cells were excluded using fixable viability dyes (eBioscience) and the Foxp3 staining buffer set (eBioscience) was used for detection of intracellular and intranuclear antigens.

### Histology and Stereology

Implantation sites of pregnant females at gd9.5 were fixed in formalin, embedded in paraffin and then cut in serial sections of 7 µm. Sections at 49 µm intervals were stained with hematoxylin and eosin following the standard procedure and stained for smooth muscle actin as previously described (70, 71). To determine tissue volumes from the serial sections, the Cavalieri method was used as previously described (70, 71). A nanozoomer slide scanner (Hamamatsu) and NDP view2 software (Hamamatsu) were used to scan the slides and quantitate the lumen area and vessel-to-lumen ratios.

### Statistical Analyses

Normally distributed, independent, and unmatched data were analyzed with two-tailed, unpaired Student’s t-tests. Comparisons of cell populations within the same individual (e.g. two NK cell subsets within the same organ) were analysed with two-tailed, paired Student’s t-tests. Data clustered by litter (fetal/placental weights) that violate the assumption of independence of data points were analyzed using a mixed-model approach taking into account the fixed effect of parental cross and the random effect of litter variability (38, 48). Categorical data were analyzed using Fisher’s exact test. *p* < 0.05 was taken as statistically significant for all tests. Analyses were performed using GraphPad Prism, GraphPad QuickCalcs, and IBM SPSS.

### Cytokine Tissue Quantification

Tissue from pregnant females at gd10.5 were snap frozen. 150mg tissue from the antimesometrial side of implantation sites were added to 1mL lysis buffer and processed with a tissue homogenizer. After centrifugation of the homogenate, the supernatant was collected and analysed with MesoScale Ultra Sensitive platform as described previously (69). Total protein concentration was determined by Bradford assay (Pierce).

## Author Contributions

JK and FC conceived the study and JK, DD, DH, NS, AS, and FC designed, performed, or analysed experiments. BM and RB generated RNA seq data as in ref. 50 and data are shown in **Figures S2**, **S7**. DD, JK, and FC wrote the manuscript. All authors critically read the manuscript.

## FUNDING

This work was supported by the Cambridge NIHR BRC Cell Phenotyping Hub and by grants from the Wellcome Trust (094073/Z/10/Z), the MRC (MR/P001092/1) and the Centre for Trophoblast Research.

## Conflict of Interest

The authors declare that the research was conducted in the absence of any commercial or financial relationships that could be construed as a potential conflict of interest.

## Publisher’s Note

All claims expressed in this article are solely those of the authors and do not necessarily represent those of their affiliated organizations, or those of the publisher, the editors and the reviewers. Any product that may be evaluated in this article, or claim that may be made by its manufacturer, is not guaranteed or endorsed by the publisher.
