# Supplementary material for: Beyond Maternal Tolerance: Education of Uterine Natural Killer Cells by Maternal MHC Drives Fetal Growth
Source: Front Immunol. 2022 May 10;13:808227. doi: 10.3389/fimmu.2022.808227 (PMC9127083; doi:10.3389/fimmu.2022.808227)
Supplement: Supplementary file 1 [file DataSheet_1.docx]

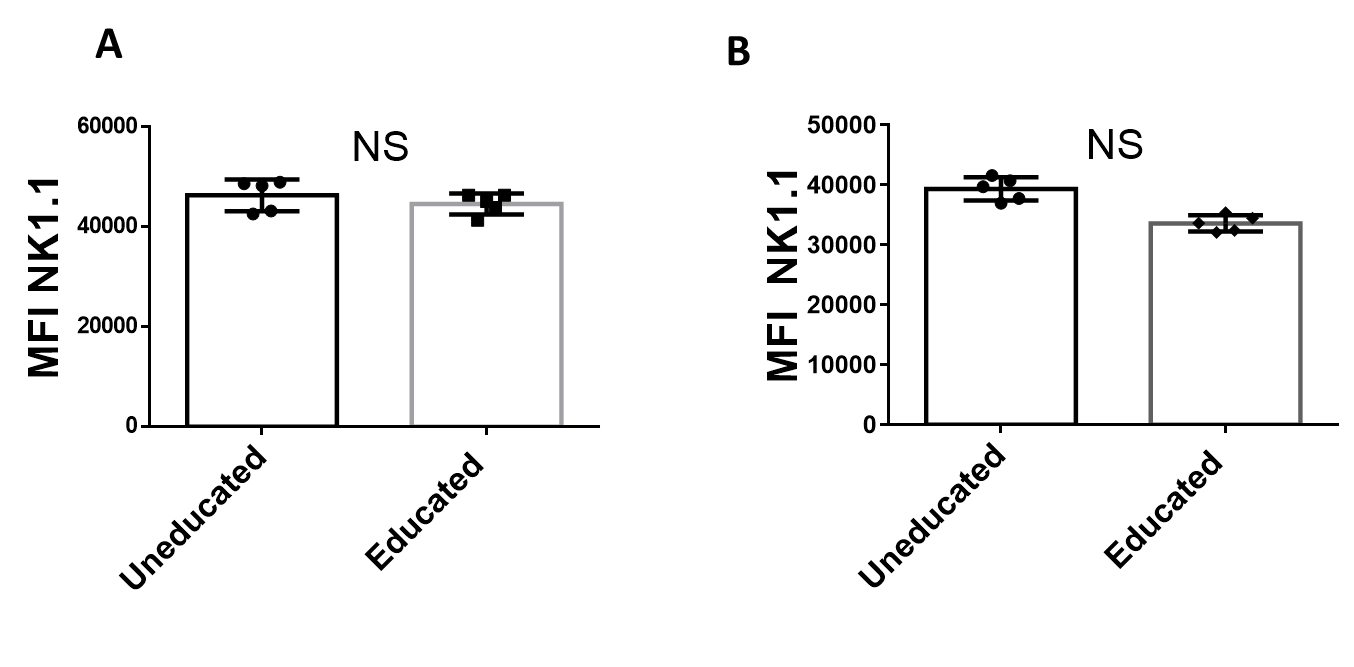


**Figure S1 :** **Educated and uneducated NK cell subsets express equivalent amount of surface NK1.1.** Comparison of NK1.1 surface expression in educated and non-educated NK cell subsets in the spleen (A) and uterus (B) of B6 mice (n=5) at gestation day 10.5. Educated and uneducated NK cells were defined as shown in Figure 3C. Statistics were calculated with a two-tailed paired Student’s t test , NS p>0.05.


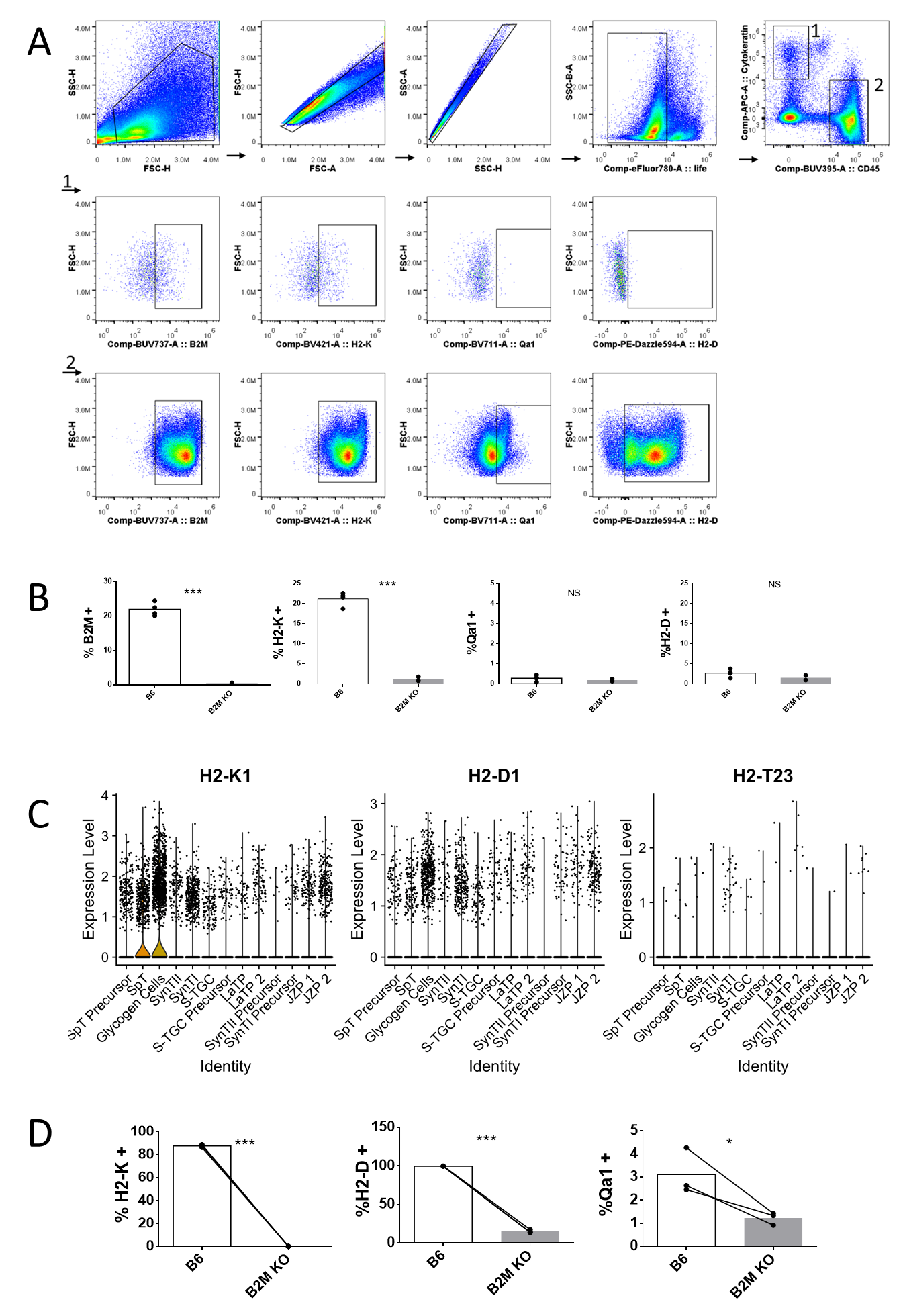


**Figure S2: Change in MHC-I molecules surface expression in the absence of B2M**.

A) Representative gating strategy used to analyse MHC-I molecule surface expression in gd12.5 pregnant mice trophoblast cells (1) (Viable singlet cells, cytokeratin positive and CD45 negative). Lymphocytes population (2) (Viable singlet cells, cytokeratin negative and CD45 positive) is shown for positive control expression. Gates were set using appropriate FMO and isotypes controls.

B) Comparison of the percentage of trophoblast cells positive for the indicated markers in B6 (n=4) and B2MKO (n=2) mice at gestation day 12.5.

C) Transcript expression level of the indicated markers in trophoblast cells from B6 mice. Cell subsets are as follows: Data generated in Marsh and Blelloch, 2020. *H2-K1* is the transcript for H-2K, *H2-D1* is the transcript for H-2D and *H2-T23* is the transcript for Qa-1. Trophoblast cell identity is as follows: Sp T Precursor: Spongiotrophoblast precursors; Sp T: Spongiotrophoblast; Glycogen Cells; Syn TII: Syncytiotrophoblast layer-II; Syn TI: Syncytiotrophoblast layer; S-TGC: Sinusoidal trophoblast giant cells; S-TGC Precursor: Sinusoidal trophoblast giant cells precursor; LaTP: Labyrinth trophoblast progenitor; LaTP2: Labyrinth trophoblast progenitor-2; Syn TII Precursor: Syncytiotrophoblast-II precursor; Syn TI Precursor: Syncytiotrophoblast recursor; JZP 1: Junctional zone precursor 1; JZP 2: Junctional zone precursor 2.

D) Comparison of the percentage of splenic cells positive for the indicated markers in B6 and B2MKO mice, n=3 mice per group.

Statistics in B and C were calculated with an unpaired two-tailed Student’s t-test, significance as follows: NS p>0.05, * p<0.05, ** p<0.01, ***p<0.001.


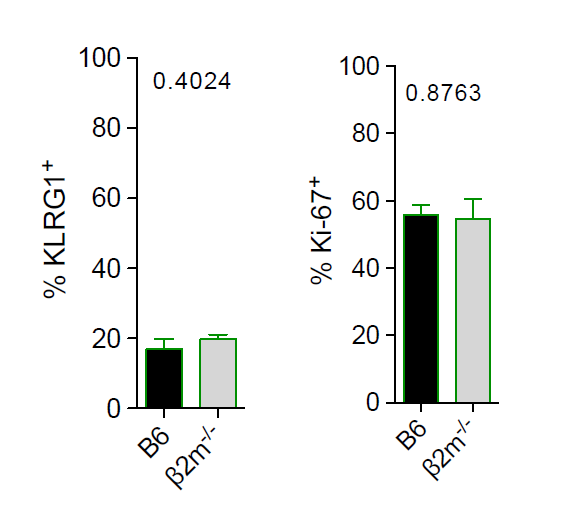


**Figure S3: No apparent change in uILC1 phenotype in the absence of maternal MHC class I expression**. Phenotypic assessment uILC1 for KLRG1 (left) which marks terminal maturation and correlates with education in cNK cells and for Ki-67 (right) which is associated with cell proliferation. Data representative of 3 experiments with n=7 mice per group. Means ± SEM. P-values from unpaired two-tailed Student’s t-tests.


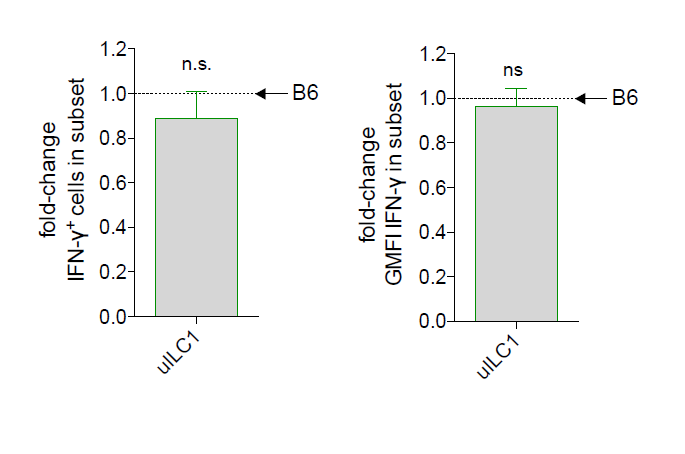


**Figure S4: Absence of maternal MHC class I does not affect uILC1 IFN-γ production.** (Left) Fold-change in fraction of subsets of uNK positive for IFN-γ compared to B6 controls. (Right) Fold-change in mean fluorescence intensity among IFN-γ+ uILC1 compared to B6 controls. Data representative of three independent experiments with n=5-8 mice per group. Means ± SEM. P-values from unpaired two-tailed Student’s t-tests; ns: not significant. GMFI: geometric mean fluorescence intensity.


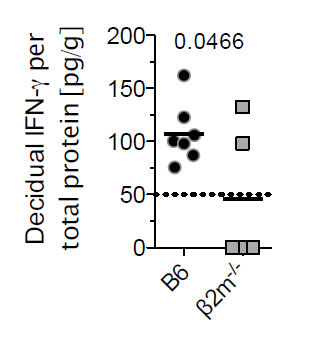


**Figure S5: Quantification of tissue IFN-γ**. IFN-γ in pooled biopsies of mesometrial sides of gd10.5 implantation sites. Data normalised to total protein concentration. Dashed line demarcates detection threshold. Data representative of n = 5-7 litters. P-values from unpaired Student’s t-tests.


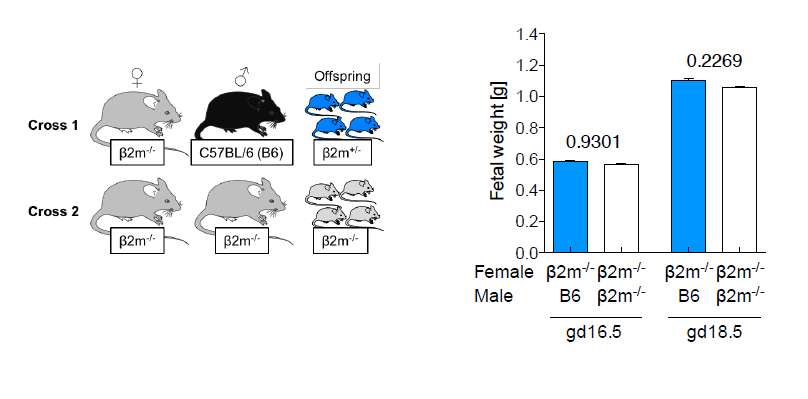


**Figure S6: Lack of placental MHC class I expression does not rescue the phenotype in *β2m^-/-^* females .** Mating strategy to control for placental/fetal expression of paternal MHC class I. Comparison of fetuses with or without placental/fetal MHC class I surface expression, carried by isogenic females without MHC class I surface expression. n=46-68 fetuses from 7 – 9 litters per group. P-value from a mixed model analysis taking the clustering of observations by litter into account.


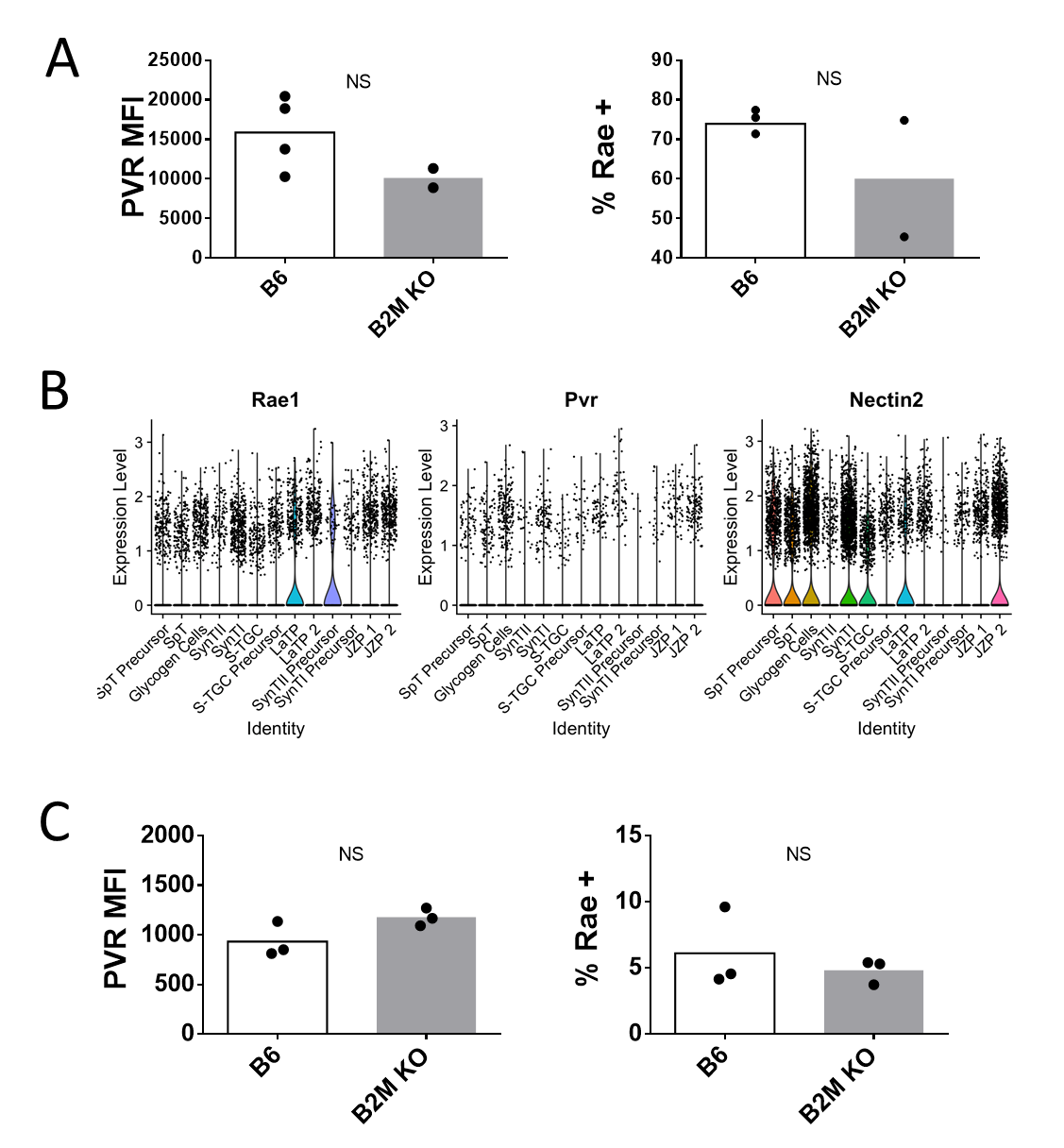


**Figure S7**: **Surface expression of NK cell activation markers is equivalent in B6 and B2MKO mice**.

A) Comparison of the percentage of trophoblast cells positive for the indicated markers in B6 (n=3) and B2MKO (n=2) mice, at gestation day 12.5.

B) Transcript expression level of the indicated markers in trophoblast cells from B6 mice. Data generated in Marsh and Blelloch, 2020. Trophoblast cell identity is as follows: Sp T Precursor: Spongiotrophoblast precursors; Sp T: Spongiotrophoblast; Glycogen Cells; Syn TII: Syncytiotrophoblast layer-II; Syn TI: Syncytiotrophoblast layer; S-TGC: Sinusoidal trophoblast giant cells; S-TGC Precursor: Sinusoidal trophoblast giant cells precursor; LaTP: Labyrinth trophoblast progenitor; LaTP2: Labyrinth trophoblast progenitor-2; Syn TII Precursor: Syncytiotrophoblast-II precursor; Syn TI Precursor: Syncytiotrophoblast recursor; JZP 1: Junctional zone precursor 1; JZP 2: Junctional zone precursor 2.

C) Comparison of the percentage of splenic cells positive for the indicated markers in B6 and B2MKO mice, n=3 mice per group.

Statistics in A and C were calculated using an unpaired two-tailed Student’s t-test , significance is indicated as follow : NS p>0.05, * p<0.05, ** p<0.01, ***p<0.001. See Supplementary Figure S3 for representative gating strategy of trophoblast cells.


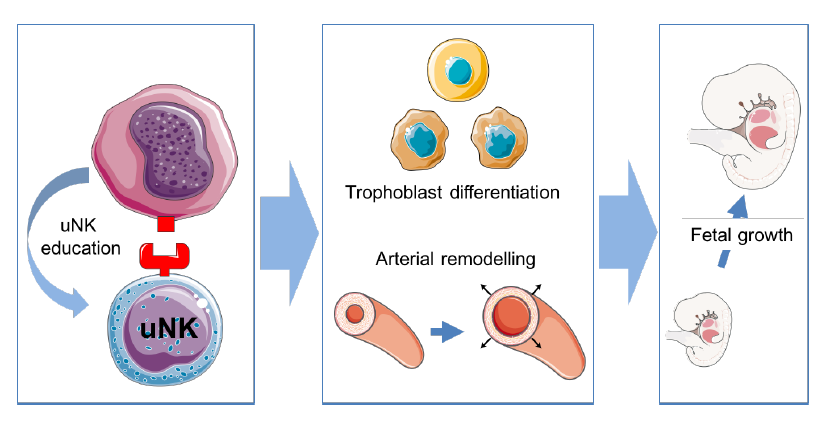


**Figure S8**: **Model**. uNK cells educated by maternal MHC have a higher functional responsiveness and are important for normal trophoblast differentiation and arterial remodelling. Ultimately, they contribute to normal fetal growth.
